# Long-term outcomes after using retrievable vena cava filters in major trauma patients with contraindications to prophylactic anticoagulation

**DOI:** 10.1007/s00068-022-02074-y

**Published:** 2022-08-27

**Authors:** Kwok M. Ho, Priya Patel, Jenny Chamberlain, Sana Nasim, Frederick B. Rogers

**Affiliations:** 1 Medical School, University of Western Australia, ICU, Royal Perth Hospital, 2nd Floor, North Block, Perth, WA Australia; 2 School of Veterinary and Life Sciences, Murdoch University, Perth, WA Australia; 3 Department of Intensive Care Medicine, Royal Perth Hospital, Perth, WA Australia; 4 Department of Surgery, Royal Perth Hospital, Perth, WA Australia; 5 Department of Surgery, Regions Hospital, St. Paul, MN 55101 USA

**Keywords:** Complications, Costs, Prevention, Vena cava filters, Venous thromboembolism

## Abstract

**Purpose:**

To investigate the long-term outcomes of using vena cava filters to prevent symptomatic pulmonary embolism (PE) in major trauma patients who have contraindications to prophylactic anticoagulation.

**Methods:**

This was an a priori sub-study of a randomized controlled trial (RCT) involving long-term outcome data of 223 patients who were enrolled in Western Australia. State-wide clinical information system, radiology database and death registry were used to assess long-term outcomes, including incidences of venous thromboembolism, venous injury and mortality beyond day-90 follow-up.

**Results:**

The median follow-up time of 198 patients (89%) who survived beyond 90 days was 65 months (interquartile range 59–73). Ten patients (5.1%) died after day-90 follow-up; and four patients developed venous thromboembolism, including two with symptomatic PE, all allocated to the control group (0 vs 4%, *p* = 0.043). Inferior vena cava injuries were not recorded in any patients. The mean total hospitalization cost, including the costs of the filter and its insertion and removal, to prevent one short- or long-term symptomatic PE was A$284,820 (€193,678) when all enrolled patients were considered. The number of patients needed to treat (NNT = 5) and total hospitalization cost to prevent one symptomatic PE (A$1,205 or €820) were, however, substantially lower when the filter was used only for patients who could not be anticoagulated within seven days of injury.

**Conclusion:**

Long-term complications related to retrievable filters were rare, and the cost of using filters to prevent symptomatic PE was acceptable when restricted to those who could not be anticoagulated within seven days of severe injury.

**Supplementary Information:**

The online version contains supplementary material available at 10.1007/s00068-022-02074-y.

## Introduction

The use of retrievable vena cava filters to prevent symptomatic pulmonary embolism (PE) in major trauma patients who have contraindications to prophylactic anticoagulation remains contentious [1]. In addition to substantial detrimental effects on patients’ short- and long-term functional and psychosocial capacity [2, 3], symptomatic PE after major trauma is also costly with an attributable hospitalization cost of over A$40,000 [4]. On the other hand, vena cava filters are expensive [5] and associated with serious complications [6]. Although a number of long-term complications of vena cava have been well described by observational studies [6, 7], whether these complications are related to the issue of confounding when the filters were used in a sicker cohort of trauma patients and left in situ for a prolonged period of time, is uncertain. Data on long-term complications of using vena cava filters to prevent PE in comparison to a comparable cohort of severe trauma patients who do not receive a filter in a randomized controlled trial (RCT) setting have not been reported.

As part of our plan to establish the evidence base and safety of using vena cava filters to prevent symptomatic PE in major trauma patients who have contraindications to prophylactic anticoagulation [8, 9], an a priori sub-study was planned to assess the long-term safety of using prophylactic vena cava filters in major trauma patients. We report the long-term outcomes of the enrolled patients beyond day-90 follow-up in this study, including incidences of venous thromboembolism, venous injury and mortality.

## Methods

The da Vinci trial was an RCT (Australian New Zealand Clinical Trials Registry number: ACTRN12614000963628) assessing the effectiveness of early placement of vena cava filters in reducing mortality and symptomatic PE in severe trauma patients who had an Injury Severity Score (ISS) of over 15 and contraindications to prophylactic anticoagulation within 72 hour of trauma admission [8, 9]. All competent patients provided written informed consent before enrollment. For those who were not competent to provide consent, their next of kin agreed to enrollment and signed an acknowledgment document; patients provided written informed consent after they regained competence. The patient consent included allowing access to long-term health outcomes beyond day-90 follow-up using data from state health information systems.

The current a priori sub-study assessed the incidences of venous thromboembolism, venous injury and mortality beyond day-90 follow-up as well as the financial cost needed to prevent one short- or long-term symptomatic PE. A total of 240 patients were enrolled in the trial, but only 223 patients were enrolled in Western Australia with complete financial data and long-term outcome data and they were the subjects of this study. State-wide clinical information systems, including hospital discharge summaries and surgical operation records, radiology database and death registry, were used to assess long-term outcomes of the study patients beyond day-90 follow-up. Important complications related to vena cava filters, such as stricture of deep veins leading to vena cava syndrome, chronic venous insufficiency, requiring external stent or repair by direct anastomosis, and venous thromboembolic events, were specifically targeted (Online supplementary sTable 1). A governmental centralized health process mandating retrieval of vena cava filters within 6–8 weeks by interventional radiologists who insert these devices had been implemented before initiation of the trial. The da Vinci trial started and completed the enrollment in May 2016 and December 2018, respectively; the censor date for the current long-term outcome study was March 4, 2022. In this study, short-term symptomatic PE was defined as those that occurred within 90 days of enrollment, and for those that occurred beyond 90-day follow-up, it was defined as long-term symptomatic PE.

Categorical and non-normally distributed continuous variables were analyzed by chi-square and Mann–Whitney tests, respectively. Log-rank test and Kaplan–Meier curves were used to compare the incidence of symptomatic PE between the two groups since enrollment and including the first 90 days. All hospitalization cost data were drawn from the hospital finance department and all resources were standardized to 2020 Australian dollars, using financial resource data such as Australia’s Medicare Benefits Scheme (MBS) for medical procedures, the Australian Red Cross cost data for blood products, and the Pharmaceutical Benefits Scheme for pharmaceuticals. In this study, one Australian dollar was converted to 0.68 Euro dollar (€) when reporting the cost in Euro currency. The details of our economic costing were described in our economic analysis sub-study [5].

All tests were two-tailed and conducted using SPSS for Windows (version 24.0, IBM, USA, 2015) and MedCalc^®^ (Statistical Software version 20.007, MedCalc Software Ltd, Ostend, Belgium; https://www.medcalc.org; 2021). A *p* value < 0.05 without adjusting for multiple testing was considered as significant in this study.

## Results

Of the 223 patients analyzed in this study, 198 (89%) survived beyond day-90 follow-up. Their median follow-up time after enrollment was 65 months (interquartile range 59–73). Two patients in the filter group did not get the filters and two patients in the control group received the filters due to clinical indications after trial treatment allocation. For those allocated to the filter group, the median duration of the filters left in situ was 55 days (interquartile range 18–102, range 4–234 days; 33 patients had their filters removed after day-90 follow-up mostly due to difficulty to access the neck veins). All patients including those allocated in the control group who had a filter placed had their filters removed eventually. The baseline characteristics of the patients, including age, body mass index, injury severity score and injury patterns were not significantly different between the two groups (Table 1).

**Table 1:** Characteristics of the da Vinci trial patients included in the current long-term outcome study (*N* = 198)

Variable	Filter group (*n* = 99)	Control group^#^ (*n* = 99)	*P* value^#^
Age, years (IQR)	33 (25–53)	37 (28–55)	0.298
Male, no. (%)	74 (75)	77 (78)	0.739
Body mass index (IQR)	26 (23–30)	27 (24–30)	0.580
Injury severity score (IQR)	27 (21–33)	26 (22–36)	0.357
Trauma embolic severity system score (IQR)	8 (5–10)	9 (6–10)	0.616
Traumatic brain injury with intracranial hematoma, no. (%)*	58 (59)	48 (49)	0.200
Spinal injury with neurological deficits, no. (%)*	26 (26)	28 (28)	0.873
Active bleeding requiring more than 6 units of red blood cells transfusion within 24 h of admission, no. (%)*	4 (4)	10 (10)	0.330
Lower limb fractures, no. (%)	14 (14)	22 (22)	0.197
Pelvic fractures, no. (%)	13 (13)	19 (19)	0.334
Intensive care unit length of stay, days (IQR)	8 (2–11)	6 (0–12)	0.437
Hospital length of stay, days (IQR)	25 (16–37)	20 (11–38)	0.105
Prophylactic anticoagulant initiated within 7 days of injury, no. (%)	69 (70)	73 (74)	0.636
Duration of the vena cava filters left in situ, days (IQR when available) (a filter was inserted for 97 and 2 patients in the filter and control groups, respectively)	55 (18–102)	98 and 112	0.330
Total dose of unfractionated heparin, units as an inpatient within 90 days of enrollment (SD)	235,598 (288,350)	195,046 (250,840)	0.371
Mean daily dose of unfractionated heparin, units including inpatient days without heparin within 90 days of enrollment (SD)	2,618 (3,204)	2,167 (2,787)	0.371
Total dose of enoxaparin, mg as an inpatient within 90 days of enrollment (SD)	1,634 (2,636)	1,314 (1,398)	0.586
Mean daily dose of enoxaparin, mg including inpatient days without enoxaparin within 90 days of enrollment (SD)	18.2 (29.3)	14.6 (15.5)	0.586

*SD*, standard deviation. ^#^Mann–Whitney or chi-square test. Continuous data are in median (interquartile range [IQR]) and categorical data are numbers (with percentage)

*Representing contraindications to prophylactic anticoagulants within 72 h of trauma admission. A total of 56 patients did not receive prophylactic anticoagulant within 7 days after injury; and differences in incidence of traumatic brain hematoma (filter group: 21/30 vs control group: 15/26, *p* = 0.408), spinal injury with neurological deficits (filter group: 9/30 vs control group: 10/26, *p* = 0.578) and requiring more than 6 units of red blood cells transfusion within 24 hour of trauma admission (filter group: 2/30 vs control group: 3/26, *p* = 0.655) were not significantly different between the two groups

**Fig. 1:**
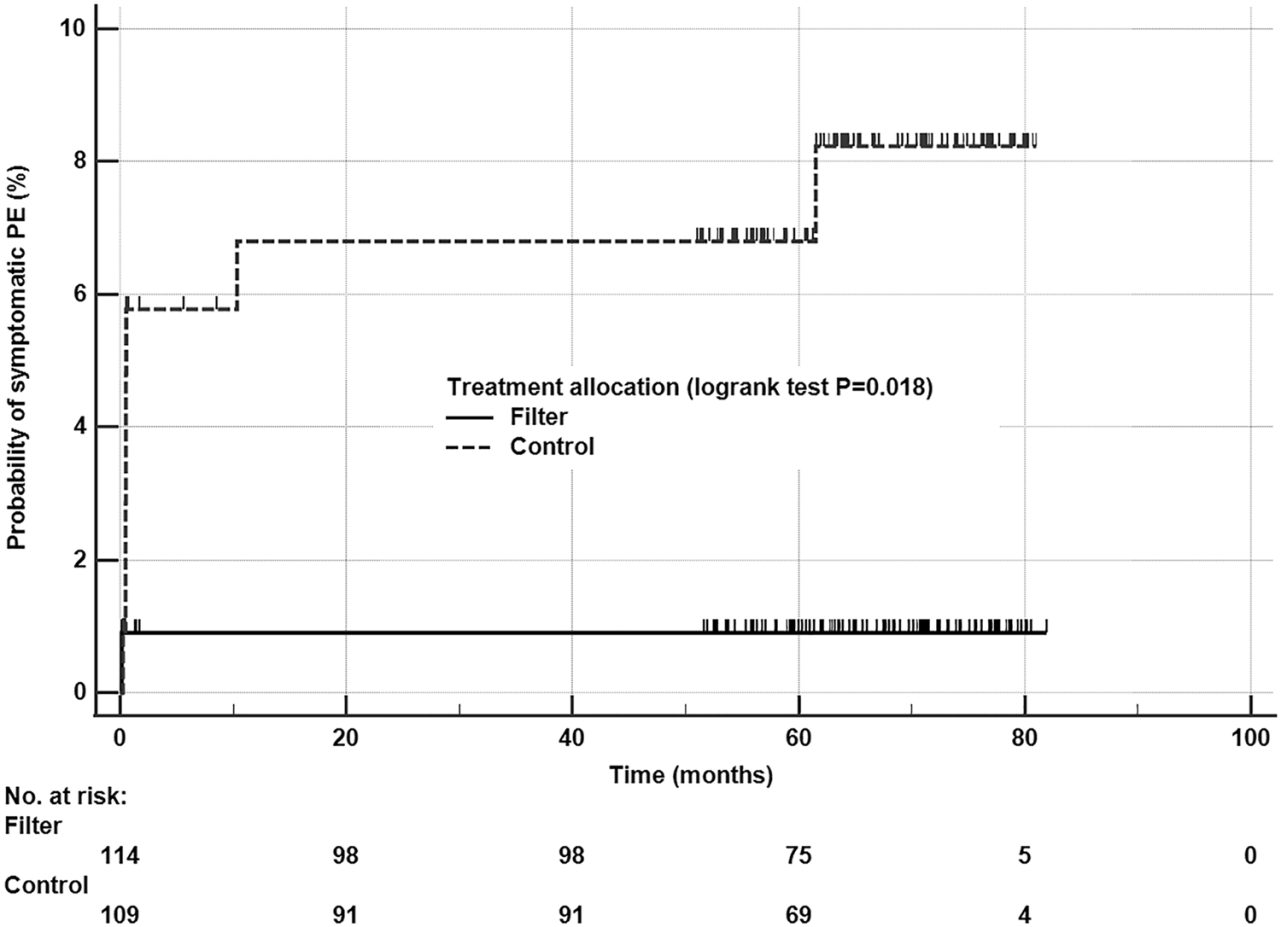
Difference in probability of developing symptomatic pulmonary embolism (PE) between the control and vena cava filter groups. NB: censored patients are indicated by the vertical lines

Ten patients (5.1%) died after day-90 follow-up and none of them were noted to die from causes related to thromboembolism. Four patients developed venous thromboembolism, all were allocated to the control group (0 vs 4%,* p* = 0.043), including two with symptomatic PE–one at 10 months after enrollment as an inpatient in a rehabilitation facility on anticoagulant prophylaxis and one at 62 months after enrollment following readmission for distal femur fractures two days before the development of PE. Both patients who had symptomatic PE beyond day-90 follow-up (in the control group) had spinal cord injury with persistent lower limb neurological deficits. One of the four patients who developed venous thromboembolism beyond day-90 follow-up died about five years after an episode of lower limb deep vein thrombosis at 5.5 months after enrollment. The two patients who had symptomatic PE did not die prior to the censor date. Overall, patients allocated to the filter group experienced a significantly reduced risk of developing symptomatic PE (Log-rank test: *p* = 0.018) (Fig. 1). Symptomatic PE from enrollment to March 4, 2022 for those who could not be anticoagulated within 7 days of injury, was significantly higher among those allocated to the control group (*N* = 72: 0/41 for the filter group vs 6/31 for the control group; *p* = 0.005) (Table 2). Inferior vena cava or other vascular injuries or operations needed to repair deep veins were not recorded in any of the trial patients during the long-term follow-up period, and there was also no significant difference in their long-term mortality between the two groups (Table 2).

**Table 2:** Long-term mortality and venous thromboembolic (VTE) outcomes of all patients with severe trauma enrolled into the da Vinci trial in Western Australia with complete data including hospitalization cost available for the study (*N* = 223)

Outcome	Filter group (*n* = 114)	Control group (*n* = 109)	*P* value^#^
All forms of VTE within 90 days of enrollment, no. (%)	22 (19.3)	15 (13.8)	0.267
Symptomatic PE within 90 days of enrollment, no. (%)	1 (0.9)	6 (5.5)	0.048
All forms of VTE beyond 90 days after enrollment, no. (%) (*N* = 198)	0 (0)	4 (4)	0.043
Symptomatic PE beyond 90 days after enrollment, no. (%) (*N* = 198)	0	2 (2)*	0.155
Symptomatic PE since enrollment with follow-up until March 4, 2022, no. (%) (*N* = 223)	1 (0.5)	8 (7.3)	0.014
Symptomatic PE since enrollment for those who could not be anticoagulated within 7 days of injury with follow-up until March 4, 2022, no. (%) (*N* = 72: 41 for the filter group, 31 for the control group)	0 (0)	6 (19.4)	0.005
Mean hospitalization cost up to day 90 after enrollment (standard deviation)** (*N* = 223), A$	140,112 (100,143)	121,124 (99,187)	0.040
Mean total hospitalization cost up to day 90 after enrollment for those who could not be anticoagulated within 7 days of injury (standard deviation)*** (*N* = 72), A$	138,280 (103,692)	132,254 (85,232)	0.941
Mortality beyond 90 days after enrollment, no. (%) (*N* = 198)	6 (6.1)	4 (4.0)	0.516

*PE* pulmonary embolism

*At 10 and 62 months and both patients had spinal cord injury with neurological deficits. ^#^By chi-square or Mann–Whitney test. **The mean total hospitalization cost, including the costs of the filter and the procedures to insert and remove the filter, to prevent one symptomatic PE was A$284,820 when all patients were considered. ***The mean total hospitalization costs, including the costs of the filter and the procedures to insert and remove the filter, to prevent one symptomatic PE was A$1,205 when the filter was used only for patients who could not be anticoagulated within 7 days of injury

The mean total hospitalization cost, including the costs of the filter and its insertion and removal, to prevent one short- or long-term symptomatic PE was A$284,820 (€193,678) when all enrolled patients were considered (Table 2). The number of patients needed to treat (NNT = 5) and total cost to prevent one symptomatic PE (A$1,205 or €820) were, however, substantially lower when the filter was used only for patients who could not be anticoagulated within seven days of injury.

## Discussion

During the median follow-up period of 65 months, we did not observe significant morbidities including an increased risk of lower limb venous insufficiency or deep vein thrombosis that could be attributed to the use of retrievable vena cava filters after major trauma compared to the control group. Venous injuries such as stricture in the vena cava after the use of retrievable vena cava filters to prevent symptomatic PE in major trauma patients were also not observed. Patients allocated to the filter group had a significantly reduced risk of symptomatic PE compared to the control group when all symptomatic PE events were considered, including those that occurred within day-90 follow-up. The cost of using the filter to prevent symptomatic PE was most acceptable when it was used for those who could not receive prophylactic anticoagulation within 7 days of injury. These results have some potential implications and require further discussion.

Although vena cava filters have been used for either primary or secondary prevention of symptomatic PE in major trauma patients for several decades, their long-term cost-effectiveness and safety outcomes in these high-risk patients remain contentious [1, 5, 10–13], primarily due to a lack of reliable data from RCTs. Our previous economic analysis based on 90-day follow-up data from patients who had contraindications to prophylactic anticoagulation within three days of trauma admission showed that the cost of using vena cava filters to prevent symptomatic PE in trauma was prohibitive (even though it was indeed effective but was not explicitly reported in our primary manuscript: 1/122 in the filter group vs 7/118 in the control group; *p* = 0.033) [9], and such strategy could only be considered cost-effective when restricted to those who had a prolonged period (> 7 days) of contraindications to prophylactic anticoagulation (€24,586 to prevent one symptomatic PE and €21,014 to gain one quality-adjusted-life-year). Our current study included follow-up data of the same cohort of patients up to a median period of 65 months and confirmed that using vena cava filters to prevent symptomatic PE was only cost-effective when used for those who had contraindications to prophylactic anticoagulation for more than seven days after injury. For such patients, the cost to prevent one symptomatic PE (€820) would be well below the incremental cost of symptomatic PE in major trauma patients [4]. As such, our recommendation on restricting the use of vena cava filters as a temporizing measure only for severe trauma patients who have ongoing contraindications to prophylactic anticoagulation beyond 5–7 days after severe injury remains unchanged [9, 14].

In this study, we did not detect significant morbidities specifically related to the use of vena cava filters such as an increased risk of lower limb deep vein thrombosis or vena cava strictures leading to chronic venous insufficiency or requiring stenting or surgical repair. When the device is used to prevent symptomatic PE including fatal PE [10], a lack of adverse long-term sequelae attributable to the use of vena cava filters in young trauma patients is reassuring and may, at least in part, contribute to its cost-effectiveness when used for those who have a prolonged period of contraindications to prophylactic anticoagulation.

It is a dilemma to decide when a retrievable filter should be removed in major trauma patients who are at high ongoing risk of developing symptomatic PE such as those who have permanent lower body neurological deficits from severe spinal injuries. The balance between increasing the risk of filter complications by leaving the filter in situ for too long and the ongoing risk of symptomatic PE can be illustrated by the two patients who had spinal injuries and developed symptomatic PE beyond day-90 after injury in the control group, including one who was still receiving prophylactic anticoagulant as an inpatient in a rehabilitation facility. Previous studies showed that lower limb deep vein thrombosis could occur in more than 20% of spinal injury patients within the first year and paraplegia increases the hazard of such event by more than four folds [15, 16]. Although the general recommendation to remove any retrievable filters that are used as a temporizing measure as soon as prophylactic anticoagulation is safely established to mitigate filter-related complications, remains sound and valid [9, 17], the decisions on whether the filters should be left in situ more than 6–8 weeks should be individualized among those who remain at high risk of developing symptomatic PE, such as those with persistent neurological deficits after spinal injury [15, 16]. The cost-effectiveness of this strategy is, however, scientifically unproven and requires further assessment by RCTs with long-term follow-up.

Finally, we need to acknowledge the limitations of this study. The duration of the filters being left in situ was relatively short in our study patients, with a median duration of 55 days, due to the state wide policy in Western Australia to remove the filters within 6–8 weeks. This is not the case in the United States where vena cava filter retrieval rates have been substantially lower (< 30%) [18]. Previous studies have shown that medical and biomechanical complications related to the filter use can increase substantially with the duration of the filter left in situ [6, 19]. As such, our findings will not be generalizable to the situations beyond short-term usage of vena cava filters to prevent symptomatic PE or centers that have a low filter retrieval rate. In this study, we only included public healthcare system health outcome data but not private healthcare data. It is possible that some patients could have long-term complications attributable to the use of the filters managed completely in a private healthcare setting, even though this would be considered exceedingly rare in trauma patients. In addition, we only included symptomatic but not asymptomatic venous thromboembolic events in this study. Although none of the ten patients who died during the follow-up period was considered to have died from PE according to their death records, we could not exclude the possibility that a subclinical or recurrent/chronic PE could have contributed to some of the deaths, as post-mortem examinations were not conducted in these patients.

The last 2 years of our follow-up period included the COVID-19 pandemic and its vaccination program in Australia. Three of the four venous thromboembolic events after day-90 follow-up were documented well before the COVID-19 pandemic, but one of the two symptomatic PE patients had the second dose of the Pfizer-Comirnaty^®^ COVID-19 vaccination 2 months before the PE (without thrombocytopenia). Whether the vaccination could have, in part, contributed to the development of symptomatic PE in this patient remains uncertain [20].

In conclusion, we did not observe any long-term complications related to the use of retrievable filters to prevent symptomatic PE in severe trauma patients who had contraindications to prophylactic anticoagulation within three days of injury, including vena cava strictures, and lower limb deep vein thrombosis and chronic venous insufficiency. The cost of using vena cava filters to prevent symptomatic PE was acceptable when restricted to those who could not be anticoagulated within seven days of severe injury. Whether the benefits will outweigh the risks by leaving the filters in situ for longer than 6–8 weeks among those who remain at high risk of developing symptomatic PE is uncertain and requires further investigation.

## Supplementary Information

Below is the link to the electronic supplementary material.

Supplementary file1 (DOCX 18 KB)
