# Supplementary material for: Long-term outcomes after using retrievable vena cava filters in major trauma patients with contraindications to prophylactic anticoagulation
Source: Eur J Trauma Emerg Surg. 2022 Aug 27;49(1):335–41. doi: 10.1007/s00068-022-02074-y (PMC9419913; doi:10.1007/s00068-022-02074-y)
Supplement: Supplementary file 1 — Supplementary file1 (DOCX 18 KB) [file 68_2022_2074_MOESM1_ESM.docx]

| **Online Supplement sTable 1**  **ICD-10AM diagnostic or ACHI procedural codes to identify complications related to arterial or venous thromboembolism and venous injury including inferior vena cava injury** |  |
| --- | --- |
| Multiple progressive thrombosis | I82.9 |
| Antepartum thro+A3:A39mbosis | O22.9 |
| Thoracic aortic thrombosis | I74.1 |
| Abdominal aortic thrombosis | I74.0 |
| Bifurcation aortic thrombosis | I74.0 |
| Saddle aortic thrombosis | I74.0 |
| Arteritis thrombosis | I74.9 |
| Basilar artery thrombosis | I65.1 |
| Common or internal carotid thrombosis | I65.2 |
| Cerebellar artery thrombosis | I66.3 |
| Cerebral artery thrombosis | I66.9 |
| Coronary artery thrombosis | I21.9 |
| Hepatic artery thrombosis | I74.8 |
| Iliac artery thrombosis | I74.5 |
| Lower limb artery thrombosis | I74.3 |
| Upper limb artery thrombosis | I74.2 |
| Unspecified limb thrombosis | I74.4 |
| Mesenteric artery thrombosis | K55.0 |
| Chronic cardiopulmonary disease including chronic cor pulmonale | I27.9 |
| Secondary pulmonary hypertension | I27.2 |
| Pulmonary artery thrombosis or embolism with acute cor pulmonale | I26.0 |
| Pulmonary artery thrombosis or embolism without specifying cor pulmonale | I26.9 |
| Renal artery thrombosis | N28.0 |
| Spinal artery thrombosis | G95.1 |
| Vertebral artery thrombosis | I65.0 |
| Brainstem artery infarction | I66.9 |
| Cerebral venous sinus thrombosis | G08 |
| Pregnancy related sinus venous thrombosis | O22.5 |
| Puerperium related sinus venous thrombosis | O87.3 |
| Femoral artery thrombosis | I80.1 |
| Iliofemoral thrombosis | I80.1 |
| Intestine thrombosis with gangrene | K55.0 |
| Pregnancy related DVT | O22.3 |
| Puerperal DVT | O87.1 |
| Puerperal superficial thrombosis | O87.0 |
| Splenic artery thrombosis | I74.8 |
| Mitral thrombosis | I34.8 |
| tricuspid thrombosis | I07.8 |
| Leg phlebitis and thrombophlebitis | I80.2 |
| Leg unspecified DVT | I80.3 |
| Leg superficial vein thrombosis or phlebitis or thrombophlebitis | I80.0 |
| Portal vein thrombosis | I81 |
| Portal vein phlebitis | K75.1 |
| Pregnancy deep thrombophlebitis | O22.3 |
| Pregnancy superficial thrombophlebitis | O22.2 |
| Puerperal superficial thrombophlebitis | O87.0 |
| Puerperal deep or pelvic thrombophlebitis | O87.1 |
| Vena cava inferior or superior thrombosis | I82.2 |
| Thrombophilia Factor V Leiden | D68.8 |
| Femoral vein embolism or thrombophlebitis | I80.1 |
| Renal vein thrombosis or embolism | I82.3 |
| Hepatic embolism | I82.0 |
| Embolism or thrombosis of unspecified vein | I82.9 |
| Phlebitis and thrombophlebitis unspecified site | I80.9 |
| Phlebitis and thrombophlebitis of other sites | I80.8 |
| Post-phlebitis syndrome | I87.0 |
| Stricture of vein including vena cava syndrome | I87.1 |
| Chronic venous insufficiency | I87.2 |
| Application of external stent to deep vein | 34833-00 |
| Repair of vena cava by direct anastomosis | 38721-01 |
| Repair of iliac vein by direct anastomosis | 33836-08 |
| Repair of other lower limb vein by direct anastomosis | 33818-13 |
| Repair of popliteal vein by direct anastomosis | 33818-12 |
| Repair of femoral vein by direct anastomosis | 33818-11 |
| Repair of other lower limb vein by graft interposition | 33821-13 |
| Repair of popliteal vein by graft interposition | 33821-12 |
| Repair of femoral vein by graft interposition | 33821-11 |
| Repair of iliac vein by graft interposition | 33839-08 |
| Repair of vena cava by graft interposition | 90217-02 |
| Vena cava bypass using vein | 34803-00 |
| Vena cava bypass using synthetic material | 34803-01 |
| Saphenoiliac vein cross leg bypass | 34806-00 |
| Saphenofemoral vein cross leg bypass | 34806-01 |
| Femoral vein bypass | 34809-00 |
| Repair of venous valve | 34818-00 |
| Vein transplantation | 34821-00 |
| Intraoperative venography | 35200-01 |
| Closed thrombectomy of inferior vena cava | 33810-00 |
| Open thrombectomy of inferior vena cava | 33811-00 |
| Closed thrombectomy of iliac vein | 33810-01 |
| Open thrombectomy of iliac vein | 33811-01 |
| Thrombectomy of femoral vein | 33812-00 |
| Thrombectomy of popliteal vein | 33812-01 |
| Thrombectomy of other large vein | 33812-04 |
| Open insertion of inferior vena cava filter | 35330-01 |
| Percutaneous insertion of inferior vena cava | 35330-00 |
| Exploration of femoral vein | 34103-08 |
| Exploration of iliac vein | 34103-07 |
| Exploration of popliteal vein | 34103-09 |
| Exploration of tibial vein | 34106-08 |
| Exploration of other vein | 34106-09 |
